# Review of Graphene-Based Textile Strain Sensors, with Emphasis on Structure Activity Relationship

**DOI:** 10.3390/polym13010151

**Published:** 2021-01-01

**Authors:** Rufang Yu, Chengyan Zhu, Junmin Wan, Yongqiang Li, Xinghua Hong

**Affiliations:** 1 College of Textiles (International Silk Institute), Key Laboratory of Advanced Textile Materials and Manufacturing Technology, Ministry of Education, Zhejiang Sci-Tech University, Hangzhou 310018, China; 201930306004@mails.zstu.edu.cn (R.Y.); cyzhu@zstu.edu.cn (C.Z.); yqqli@163.com (Y.L.); 2 Tongxiang Research Institute, Zhejiang Sci-Tech University, Tongxiang 314599, China; wwjm2001@126.com; 3 School of Materials Science and Engineering, Zhejiang Sci-Tech University, Hangzhou 310018, China

**Keywords:** textile strain sensors, graphene-based, staple fiber, staple and filament yarn, fabric

## Abstract

Graphene-based textile strain sensors were reviewed in terms of their preparation methods, performance, and applications with particular attention on its forming method, the key properties (sensitivity, stability, sensing range and response time), and comparisons. Staple fiber strain sensors, staple and filament strain sensors, nonwoven fabric strain sensors, woven fabric strain sensors and knitted fabric strain sensors were summarized, respectively. (i) In general, graphene-based textile strain sensors can be obtained in two ways. One method is to prepare conductive textiles through spinning and weaving techniques, and the graphene worked as conductive filler. The other method is to deposit graphene-based materials on the surface of textiles, the graphene served as conductive coatings and colorants. (ii) The gauge factor (GF) value of sensor refers to its mechanical and electromechanical properties, which are the key evaluation indicators. We found the absolute value of GF of graphene-based textile strain sensor could be roughly divided into two trends according to its structural changes. Firstly, in the recoverable deformation stage, GF usually decreased with the increase of strain. Secondly, in the unrecoverable deformation stage, GF usually increased with the increase of strain. (iii) The main challenge of graphene-based textile strain sensors was that their application capacity received limited studies. Most of current studies only discussed washability, seldomly involving the impact of other environmental factors, including friction, PH, etc. Based on these developments, this work was done to provide some merit to references and guidelines for the progress of future research on flexible and wearable electronics.

## 1. Introduction

The graphene-based textile strain sensor, pertaining to flexible and wearable strain sensors, is a smart material comprising the graphene, which is effectively able to sense the strain and stress. Sensing refers to the phenomenon in which the electrical resistance [1] and capacitance [2] of a material changes with the strain, etc. [3,4]. Flexible and wearable sensors have been greatly favored, mainly due to their wide applications including electronic skin [5,6,7], human-machine interfaces [8,9], human activities monitoring [10,11], intelligent robots [12], and human health detection [13,14]. Strain sensors possess the characteristics of high sensitivity, good flexibility, and good stretchability [15]. These capabilities are rendered by the embedding of devices and the use of conductive materials. Conventional strain sensors based on metal foils or semiconductors achieve good performance in terms of sensitivity. However, they are generally rigid, high weight, and have poor stretchability (<5%), which means they are not truly flexible and wearable strain sensors [8,16,17].

**Figure 1:**
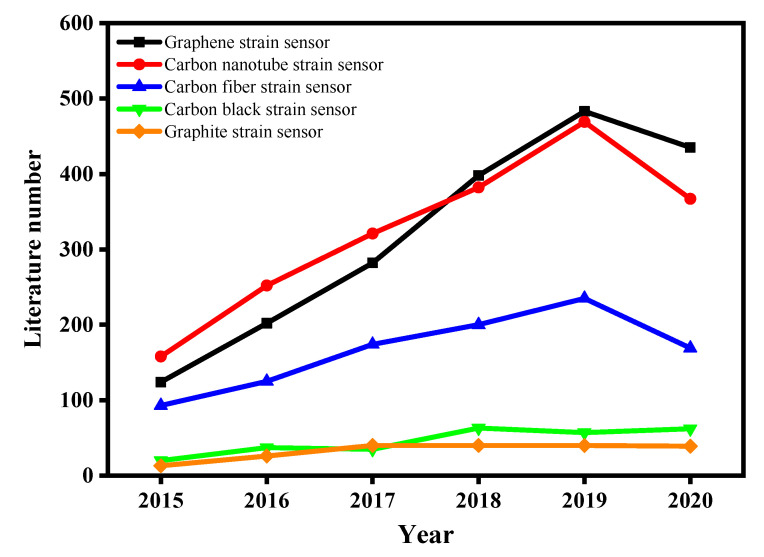
The development trend of carbon-based strain sensors in recent five years.

Various carbon materials [18], such as graphite [16,19,20], carbon black [21,22], carbon fiber [23], carbon nanotubes [24,25,26], and graphene [27,28,29,30], have been used to fabricate wearable strain sensors in recent years. Especially, graphene has been extensively studied for strain sensors due to its outstanding mechanical properties and conductivity, as shown in Figure 1. Graphene is a two-dimensional carbon nanomaterial, which is composed of a hexagonal honeycomb-like crystal lattice structure formed by sp^2^ hybridization carbon atoms [31,32,33,34]. Single-layer graphene was obtained by mechanical exfoliation (repeated peeling) of small mesas of highly-oriented pyrolytic graphite [35]. Structural and remarkable properties of graphene, including large surface area, prominent electrical properties, and excellent thermal and mechanical stability, make it a promising material for strain sensors [31,36,37,38,39]. Graphene oxide (GO) and reduced graphene oxide (RGO) are additional derivatives of graphene [40]. On the other hand, compared to other materials, textile materials are thin, flexible materials with a hierarchically porous structure, high surface area, and sufficient strength and tear resistance [41,42,43]. Therefore, for the achievement of wearable strain sensors with high sensitivity, good stability, and stretchability, the use of graphene to enhance functionality for textiles without the embedding of devices is remarkable.

In this review, we aim to provide valuable guidelines for the preparation of flexible and wearable graphene-based textile strain sensors and to study the structural relationship between textile strain sensors and the absolute value of GF. According to different external morphological structures and production stages of textiles, the research progress of staple fiber strain sensors, staple and filament strain sensors and fabric strain sensors in recent years is introduced. The preparation method, performance and applications of graphene-based textile strain sensors are described. In general, there are two ways for preparing graphene-based textile strain sensors with prominent sensitivity and stability. One is to prepare conductive textiles via spinning and weaving techniques [44,45,46,47,48,49,50,51,52,53]. The other is depositing graphene-based materials on the surface of textiles [17,54,55,56,57,58,59,60,61,62,63,64,65,66,67,68,69,70,71,72,73,74,75,76,77,78,79,80,81,82,83,84,85,86,87,88,89,90,91,92,93,94,95,96,97].

**Figure 2:**
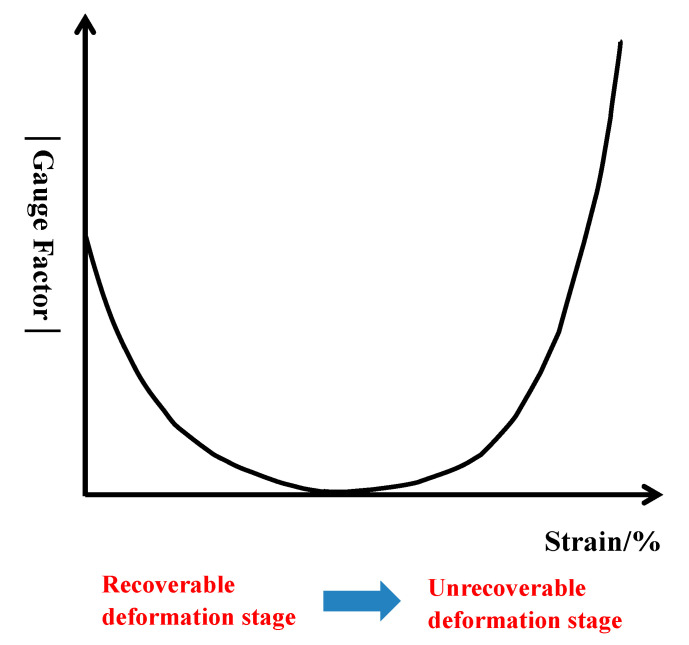
The general trend of GF absolute value of textile strain sensors according to their structure variation.

When the graphene-based strain sensor is stretched by strain, its structural changes can be roughly divided into two processes. One is recoverable deformation when the textile sensor is subjected to a small strain; the other is irreversible deformation when the textile sensor is subjected to a large strain. Therefore, the GF absolute value of the textile strain sensor can be divided into two trends according to its structural changes, as shown in Figure 2. When a small strain is applied, its GF value decreases with the increase of strain, which may be ascribed to the decrease of contact point growth rate between fibers. When the strain exceeds the allowable range of fibers (refers to the natural extension of the fibers), the conductive networks attached to the single fiber surface are destroyed and rebuilt, leading to the sharp increase of GF. Cracks in the body of the material should be noted. Under huge strain, the sensing material will crack or even fracture, leading to the infinite increase of GF.

Graphene-based textile strain sensors are mainly used to sense a wide range of motions, from small (breathing, facial expressions, vocal sounds, coughing, and human pulse, etc.) to large (human limbs) [45,46,50,51,52,57,58,59,61,62,64,65,66,68,70,71,72,76,78,79,80,83,84,85,86,87,88,91,94,95,96,97]. Based on these developments, we summarize challenges and development prospects of graphene-based textile strain sensors, providing meaningful guidance and direction for future research.

## 2. Performance Factors

Textile strain sensors can be divided into resistive, capacitive, piezoelectric, triboelectric, and optical (Fiber Bragg Grating) [98]. The resistive type is widely used in textile strain sensors because it holds the superiorities of large measurement range, simple device structure, and high sensitivity [99], which is also the main focus of this review. The performance of textile strain sensors is generally evaluated from the aspects of GF, sensing range, long-term stability, and response time [100,101].

Sensitivity is the most important property of strain sensor, which is usually expressed by GF. GF is the relative resistance that changes with strain, which is expressed in Equation (1).

(1) $$\mathrm{GF}\;=\;\frac{∆R/R_{0}}{\varepsilon }$$

In the equation, *R*_0_ is the resistance of the sensor at the initial (unstrained) state, ∆*R* is the difference between the resistance (*R*) at the stretched state and the resistance (*R*_0_) at the initial state, and *ε* indicates the mechanical strain. Therefore, the strain range of GF values should be indicated. The larger the GF, the more sensitive the strain sensor is. The resistance changes reversibly and irreversibly under the strain. The reversible change of resistance is related to strain sensing, so the strain used in the test should not be too large. Similarly, linearity is true within a certain range of deformation. In addition, the response time of sensors to the strain can also reflect the sensitivity.

The sensing range refers to the range of strain that can be detected by sensors, and it is one of the reference factors for sensors application. Stability refers to the performance of the textile strain sensor that can recover to the initial state after being stretched for many times. In the process of practical application, the textile strain sensor is bound to be stretched and worn for many times, so the stability is very important for it. Furthermore, textile strain sensors for flexible and wearable electronics should possess excellent comfort, light weight, and durability.

## 3. Preparation Methods and Performance Evaluation

From fibers to fabrics, textiles possess many unique properties including durability, conformability, deformability, breathability, and washability [68]. Therefore, the textile is regarded as an ideal material for developing strain sensors with high sensitivity and stability, although the conductivity and electrochemical properties of textile materials are poor [42]. According to production stages and the morphological structure of textiles, the preparation methods and performance of staple fiber strain sensors, staple and filament yarn strain sensors, and fabric strain sensors are discussed.

### 3.1. Graphene-Based Fiber and Yarn Strain Sensors

Fiber and yarn are widely used as sensor accessories applied to strain sensors, owing to their flexibility, adjustability, wide applicability, and low cost. They can be designed as strain sensors combining with graphene-based materials. The research of graphene-based fiber and yarn strain sensors is introduced in detail in the following sections.

#### 3.1.1. Staple Fiber Strain Sensors

**Figure 3:**
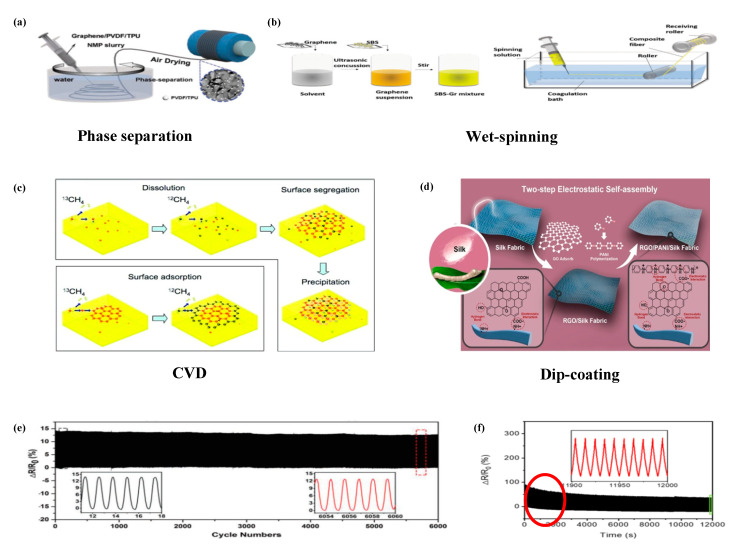
Fabrication and performance of graphene-based textile strain sensors. (**a**) Schematic illustration showing the fabrication of PGFs via a phase separation method. “Adapted with permission from reference [57]. Copyright [2019], Advanced Functional Materials”. (**b**) Schematic illustration for preparing poly (styrene-butadiene-styrene)/graphene composite fibers through a wet-spinning method. “Adapted with permission from reference [45]. Copyright [2020], Macromolecular Materials and Engineering”. (**c**) Durability of the PGFs strain sensors after 6000 cycles at a 1% strain stretch and release. “Adapted with permission from reference [57]. Copyright [2019], Advanced Functional Materials”. (**d**) Stability of the rGOFF strain sensor under a repeated applied strain of 20.0%. “Adapted with permission from reference [46]. Copyright [2019], ACS Applied Materials & Interfaces”. (**e**) Schematic diagram of CVD method. “Adapted with permission from reference [107]. Copyright [2009], Nano Letters”. (**f**) Schematic diagram of dip-coating method. “Adapted with permission from reference [108]. Copyright [2020], ACS Applied Materials & Interfaces”.

Fibers are the most primitive state of textiles, which can be divided into natural fibers and chemical fibers according to the materials. There are a great variety of fibers with different characteristics. For example, silk fibers have high tensile strength, toughness, and excellent elasticity [102]. Cotton fibers possess numerous advantages, such as easy processing, relatively low cost, and good strength and chemical resistance [103]. Polyester fibers have high strength, good chemical stability, and suitable extension and rebound performance [104]. These outstanding properties make fibers an ideal material for preparing strain sensors. Nevertheless, most fibers, such as natural fibers (cotton, silk, wool, etc.), polymer fibers (polyurethane, polyester, polyamide, etc.) and so on, are typically not capable of electrical conductivity. Even among conductive fibers (such as graphene fibers and RGO fibers), most of them have poor strength, low elongation and elasticity, and are fragile under external force [105,106]. These shortcomings hinder their promising applications in strain sensors. Therefore, some techniques are used to eliminate these shortcomings, with the purpose of fabricating fiber strain sensors. For instance, Yin et al. [64] fabricate a wearable sensor through dipping cellulose acetate fiber (CAF) bundles into the as-prepared RGO solution. Huang et al. [57] manufacture porous graphene fibers (PGFs) decorated with nanoballs via a facile phase separation method (Figure 3a). Wang et al. [44] fabricate a strain sensor based on conductive poly(styrene-butadienestyrene)/few layer graphene (SBS/FLG) composite fiber through wet-spinning method. Li et al. [45] adopt a simple wet-spinning to prepare a composite fiber-based strain sensor (Figure 3b).

Various methods for the fabrication of fiber strain sensors, including chemical vapor deposition (CVD) (Figure 3c) [55,56], phase separation [57], dip-coating (Figure 3d) [64,65,66,69], and spinning [44,45,46,47,48,49,50] have been extensively investigated. Preparation methods have important effects on properties of strain sensors, especially in sensitivity. The strain sensor prepared by dipping method possesses a wide sensing range (0.05–75%), but it owns a low sensitivity with a GF of −8.9 in lower than 5% strain [64]. The PGFs fabricated by a phase separation method show relatively high gauge factors (51 in 0–5% and 87 in 5–8%) [57]. Jiang et al. [46] employ wet-spinning to prepare a flexible sensor with an ultrahigh gauge factor (1668). Li et al. [50] manufacture a RGO nanosheet-wrapped thermoplastic polyurethane (TPU) fibrous mat through electrospinning, which exhibits great sensitivity (GF of ~50 at a strain of 50%).

The properties of various graphene-based staple fiber strain sensors of prior works are summarized in Table 1, and the attributes listed include type, main materials, fabrication method, GF, sensing range, stability, response time, and application. The following points are worth noting:

(1) Relationship between GF absolute value and structure.

With the increase of strain range, GF increases [44,45,57], which is due to the destruction and reconstruction of the conductive networks on the fiber surface. When the fiber is stretched by a small external force, a small deformation occurs, resulting in disconnection of the conductive networks contacts, and GF slowly increases. When a higher strain is applied, the conductive networks of the fiber break and irreversible damage occurs, leading to a sharp change in its resistance and a rapid rise in GF.

(2) Stability.

Stability of the graphene-based fiber sensor depends on the number of stretching cycles and the strain. Suppose stability can be defined as the number of stretching cycles multiplied by strain. The larger the value is, the better stability of the strain sensor will be. For instance, the PGFs show great durability (over 6000 cycles at 1% strain) (Figure 3e) [57]. Jiang et al. [46] prepare a flexible sensor with a great durability and stability (within 1200 cycles under a strain of 20%), as illustrated in Figure 3f. In addition, it can be found that the stability of the fiber sensor fluctuates within a certain range during the high strain stretching cycles, which may be caused by the recombination of the conductive networks. This does not affect the stability of fiber strain sensors.

#### 3.1.2. Staple and Filament Yarn Strain Sensors

Yarns are a kind of textile with a certain fineness processed from various fibers. They have a higher strain range than fibers thanks to inter-fiber slip, which comes from twisting without chemical bonding. Furthermore, staple and filament yarns possess soft, skin-friendly, tensile-stable characters and good biocompatibility [109]. These excellent properties make them attractive for the fabrication of strain sensors comprising graphene, etc. Wang et al. [54] fabricate poly(vinyl alcohol) coating graphene (G@PVA) core-sheath fibers filament by the use of a simple polymer coating method and chemical vapor deposition (CVD)-grown graphene nanofibers. However, the sensing range is only 16%, and a low sensitivity is observed, with a GF of ~5.02 under 1–6.3% strain [54]. Graphene and polymers can be employed as raw materials to fabricate filament yarn strain sensors via spinning technologies [51,52,53]. Yan et al. [51] fabricate composite nanofiber yarn strain sensors by electrospinning. The average GF of the sensor is >1700 under an applied strain of 2%.

Nevertheless, the method of combining graphene-based materials with yarns through dip-coating [58,59,65,66,67,68,70], layer-by-layer (LbL) assembly [60,61,62,63] is more widely used. Hamid Souri et al. [58] coat natural fiber yarns with graphene nanoplatelets (GNPs) and carbon black (CB) to obtain conductive yarns. However, it holds a low sensitivity with a GF of 5.62 under 4% strain. Cheng et al. [65] dip-coat a yarn with GO and reduced by hydroiodic acid to obtain a RGO coated filament yarn. Niu et al. [68] adopt a dip-coating method to obtain a strain sensor based on polydopamine/reduced graphene oxide/polyurethane yarn (PDA/rGO/PUY). Li et al. [60] coat graphene onto a polyurethane (PU) core via a layer-by-layer assembly method. The strain sensor possesses a high GF of 86.86 and excellent thermal stability. Besides, to further increase the sensitivity, Li et al. [61] use graphene-microsheets (GMs)/PU strain sensing yarns to prepare a flexible strain sensor, which is endowed with high sensitivity (a GF of 490.2 under an applied strain of 50%). More importantly, the strain sensing yarns can be directly woven into fabric to make a wearable and flexible strain sensing fabric. Li et al. [63] also fabricate a yarn strain sensor based on graphene nanosheets/thin gold film/graphene nanosheets/polyurethane (GNSs/Au/GNSs/PU). It possesses a high GF (~662 within 50% strain), remarkable stability, and waterproof property. In short, the properties of the strain sensors prepared by different methods are disparate.

The performance of the graphene-based staple and filament yarn strain sensor is not only related to the preparation method, but also closely related to the type of yarns. Jung Jin Park et al. [62] adopt LBL assembly technique to manufacture three types of strain sensors, made from different yarns (rubber (RY), nylon covered rubber (NCRY), and wool yarn (WY)). These yarn sensors show different sensitivities with GFs (~1800 (RY), 1.4 (NCRY), and −0.1 (WY)).

The performance of various graphene-based staple and filament yarn strain sensors are summarized in Table 2. It can be found that the GF absolute value of staple and filament yarn strain sensors present different variation trends with the increase of strain [59,65,66,68,70]. In the process of stretching, yarn deformation can be divided into two processes. One is recoverable micro-deformation. The contact point between yarns decreases with the increase of strain, leading to the decrease of GF [65,66,70]. The other is irreversible deformation that occurs when the strain exceeds the allowable range of the yarn. The conductive networks on the yarn surface are destroyed and restructured, which leads to the increase of GF [59,68].

To sum up, two methods are adopted to obtain flexible fiber and yarn strain sensors. One is to prepare conductive fiber and yarn by various spinning technologies, such as liquid crystal spinning [49], wet spinning [44,45,46,47,48], or electrospinning [50,51,52,53]. The other is to deposit graphene-based materials on the surface of fibers/yarns by CVD [54,55,56], vapor-phase polymerization [57], layer-by-layer (LbL) assembly [60,61,62,63], dip-coating [58,59,64,65,66,67,68,69,70], among others. The prepared graphene-based fiber and yarn strain sensors are endowed with good sensitivity, stability, and sensing range, but most of them act as the accessories of clothes or the independent devices, and still cannot provide satisfactory comfort [70]. Most of the graphene-based fiber and yarn strain sensors need flexible substrates such as PDMS [49,54,55,57,62,64,65], Ecoflex [58,59], or TPU [51] to protect their structure because sensing fibers and yarns are easily damaged. Designing graphene-based fibers and yarns strain sensors that are endowed with prominent sensitivity and a wide sensing range, yet satisfactory comfort remains a big challenge.

### 3.2. Graphene-Based Fabric Strain Sensors

Fabrics refer to sheet objects made of textile yarns, which have good wearing comfort and softness. However, they still have several drawbacks, including unsatisfactory mechanical properties of the stretchable substrate and poor electrical conductivity [110]. Various methods are employed to combine fabrics with graphene-based materials, with the aim of converting them into highly conductive materials. According to the different processing methods of fabrics, the preparation and properties of nonwoven, woven, and knitting fabric strain sensors are discussed in detailed.

#### 3.2.1. Nonwoven Fabric Strain Sensors

Nonwoven fabrics are kinds of fabrics that do not need spinning and weaving. They only need to arrange short textile fibers or filaments in a directional or random way [111] to form a fiber net structure, and then they are reinforced by mechanical, thermal bonding, or chemical methods. Nonwoven fabrics break through the traditional textile principle and have the advantages of low cost, fast production speed, wide applications and recyclability. Therefore, strain sensors prepared by nonwoven fabrics (NWF) as sensing substrate attract considerable attention. Qu et al. [85] employ a scalable screen-printing process for creating GO patterns onto viscose nonwoven fabrics. The sensing fabric is highly sensitive to compression, good wash fastness. A strain sensor based on RGO/polyester (PET) fabrics are manufactured via suction filtration and reduction [86]. It holds durability (150 bending cycles under 10% strain) and electrothermal property. M. Simard-Normandin et al. [73] adopt a spray coating technology to fabricate a graphene-based nonwoven fabric, which exhibits good performance in terms of sensitivity (GF of 9.43 and 9 under a strain of 20% and 1%, respectively), durability, and stability. Du et al. [71] prepare a graphene-NWF (GNWF) strain sensor by a dip-and-reduce method. The sensor possesses a negative GF of −7.1, great stretchability, and stability (up to 10,000 cycles at 1% strain). Furthermore, it can be directly integrated into clothes. The RGO/carbon nanotubes (CNTs)/NWF sensors [72] with great stability, a high GF (32.65 at 1.0% strain), and high machine washability (the resistance of RGO/CNTs/NWF textile changes from ~31 to ~ 36 kΩ after 6 washing cycles) are prepared by the same method. Liu et al. [87] adopt a dip-coating method to fabricate a strain sensor, which shows excellent performance, including an ultra-sensitivity (180 at 15% strain), good stability, and excellent self-cleaning, anti-corrosion ability, and waterproofness. 

The performance of various graphene-based nonwoven strain sensors is shown in Table 3, and the following points can be found.

(1) Preparation methods and performance.

Various methods, including screen-printing [85], suction filtration and reduction [86], spray coating [73], dip-and-reduce [71,72], dip-coating [87], etc., can be adopted to combine nonwoven fabric with graphene-based materials for making strain sensors with high sensitivity, good stability, and wide sensing range. In addition to the basic performance of the strain sensors, the as-prepared sensors also possess some other properties, like wash fastness [72,85], integrating into clothes [71], self-cleaning, anti-corrosion ability, and waterproofness [87].

(2) Relationship between GF absolute value and structure.

The crack of the materials is worth noting. Graphene-based sensors exhibit reversible and irreversible changes in resistance when subjected to strain. The reversible resistance change is relevant to strain sensing, so the strain used in the testing should not be excessive. High values of the GF may be due to the involvement of an irreversible resistance change. For example, Liu et al. [87] fabricate a strain sensor, which shows a GF of about 180 at 15% strain. Then, GF reaches up to be about 23,600 under 98% strain. This may be conducted by the crack or even fracture of the sensing material under large-strain deformation, which leads to the damage of the conductive grid and open circuit, so the GF will enlarge infinitely.

Currently, in graphene-based fabric strain sensors, the research on nonwoven fabric as the substrate is less than that on woven fabric as the substrate, which may be thanks to the poor strength of nonwoven fabric. For the nonwoven fabrics, the tensile strength is obviously lower in comparison with woven fabrics [73].

#### 3.2.2. Woven Fabric Strain Sensors

Woven fabrics are made by interlacing warp and weft yarns according to certain rules [112,113]. They are endowed with good characteristics of structural stability and washing resistance. Graphene-based materials can be attached to woven fabrics in a variety of ways, for preparing graphene-based woven strain sensors with high sensitivity and stability. Liu et al. [77] prepare graphene woven fabric (GWF) via a CVD method. GWF owns high sensitivity (GF of 223 at a strain of 3%), good stability, and low hysteresis. Liu et al. [76] also employ the same method to fabricate an elastomer-filled graphene woven fabric with a high GF (282 at 20% strain). Liu et al. use an ultraviolet picosecond laser technique to produce graphene strain sensors with high sensitivity [78].

The method of coating conductive solution or dispersion on woven fabrics is more widely used because of its simplicity and ease of operation. Yin et al. [88] make a strain sensor based on RGO woven fabrics through a dip-coating method. The strain sensor possesses a high GF (3667 within 48–57% strain), an ultrafast response time, and good stretchability, durability, and stability. He et al. [81] fabricate a highly conductive silver/graphene-coated (Ag/G-coated) cotton fabric by dipping and magnetron sputtering. Zheng et al. [82] dip cotton fabric into graphene dispersion repeatedly for obtaining a strain sensor (CFSS) with good sensitivity and stability (10,000 cycles under 30% strain). In addition, the conductive solution can also be coated on woven fabrics by spin-coating [17] and spray coating [74].

Graphene-based woven fabric strain sensors can also be achieved by a vacuum filtration method. Ren et al. [80] employ a vacuum filtration method to manufacture a strain sensor, which possesses washability (sheet resistance changes from ~0.9 to ~1.2 kΩ/sq after 10 washing cycles). Wang et al. [79] use the same method to prepare a graphene-silk fabric strain sensor with excellent GF (about 124), stability, UV-blocking, and wash fastness properties.

The performance of various graphene-based woven strain sensors reported in the literature is presented in Table 4. There are different methods to obtain graphene-based woven fabric sensors, such as CVD method [76,77], direct laser writing [78], dip-coating [81,82,83,88,89,90,91,92], spin-coating [17], spray coating [74], vacuum filtration [79,80], among others. Woven fabrics are excellent flexible substrates that can be well combined with graphene-based materials. The as-prepared strain sensors are endowed with good performance, including sensitivity, stability, sensing range, etc. Compared to staple fiber and filament strain sensors, woven fabric strain sensors have better washable resistance [80,83,91], but their sensing range is small [17,76,79], which may be because of their poor stretchability.

#### 3.2.3. Knitted Fabric Strain Sensors

Knitted fabrics are made up of loops, possessing excellent elastic, softness, and comfort [114,115,116]. Mainly thanks to the existence of the winding loops that can expand in various directions, they can be highly stretched [93]. Knitted fabrics are regarded as ideal vehicles for strain sensors [94]. Graphene-based materials can be deposited on fabrics in a variety of ways. The dip-coating method is the most common method to prepare strain sensors with good performance. Hanna Lee et al. [94] obtain a strain sensor with good sensitivity (GF of 18.24 within 40.6% strain) by a dip-coating method. But the repeatability of the sensor is seldom provided. Zhang et al. [95] fabricate a graphene textile strain sensor with negative resistance variation through a dip-coating method. The sensor displays a maximum GF of −1.7 in the strain range of 15% during x-direction stretching and −26 within an 8% strain range with y-direction stretching. Ravinder Reddy K et al. [96] use the same method to prepare the RGO polyester knitted elastic band (RGOPEB) strain sensor, which exhibits excellent performance including great sensitivity, a low detection limit, and good durability and stability. Cai et al. [97] manufacture high sensitive strain sensing fabrics (FSSFs) with washability (resistance increased from ~112 kΩ/m^2^ to ~154 kΩ/m^2^ after 8 washing cycles). Graphene-based materials can also be combined with knitted fabric via spray coating [75], pad dyeing [84], etc. Xu et al. [84] pad-dyeing wool-knitted fabrics to prepare a rGO-based strain sensor, showing high sensitivity, low hysteresis, and great stability.

The properties of various graphene-based knitted strain sensors are presented in Table 5. Graphene-based knitted fabric sensors with prominent property can be realized by dip-coating [93,94,95,96,97], spray coating [75], pad dyeing [84], etc. Thanks to the unique structure of knitted fabrics, graphene-based fabric strain sensors possess resistance anisotropy [84,95]. The sensitivity of the prepared strain sensor in x-direction and y-direction is generally different. Therefore, the stretching direction of the strain sensor can be judged according to its resistance change. It is worth noting that there are positive and negative differences in GF values of graphene-based knitted strain sensors, which may be caused by different orientations. The sensitivity of the fabric strain sensor is independent of direction.

In summary, various approaches, including dip-coating [71,72,81,82,83,87,88,89,90,91,92,93,94,95,96,97], spray coating [73,74,75], spin-coating [17], CVD [76,77], vacuum filtration [79,80], suction filtration and reduction [86], screen-printing [85], direct laser writing [78], pad dyeing [84], etc., can be used to combine fabrics with graphene-based materials, with the purpose of fabricating strain sensors. Among them, the most widely used method to prepare strain sensor is to immerse the fabric in conductive solution or dispersion solution, which utilizes the water solubility and applicability of precursor GO. Most of the as-prepared graphene-based fabric sensors hold great sensitivity, stability, sensing range, etc. In addition, the strain sensor also possesses some other properties, like wear comfort [94], wash fastness [72,80,85,97], integrating into clothes [71,79,84], self-cleaning, anti-corrosion ability [87], and waterproofness [83,87,91].

## 4. Applications

**Figure 4:**
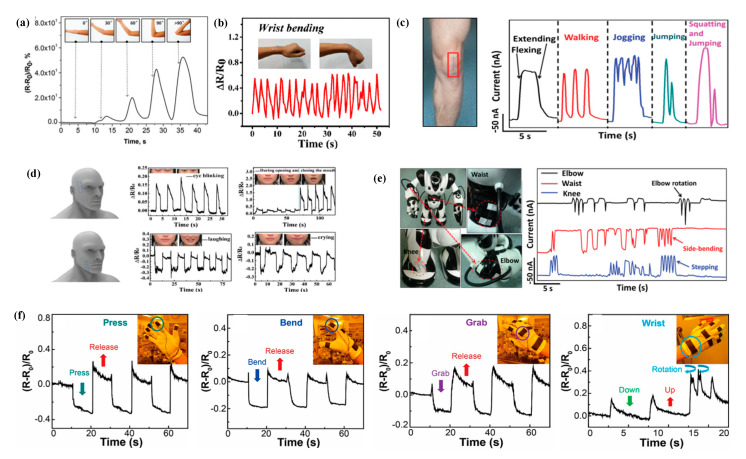
Applications of graphene-based textile strain sensors. (**a**) Relative resistance changes of the elbow joint bending. “Adapted with permission from reference [45]. Copyright [2020], Macromolecular Materials and Engineering”. (**b**) Relative resistance changes of the CFSS to wrist bending. “Adapted with permission from reference [82]. Copyright [2020], ACS Applied Materials & Interfaces”. (**c**) Responsive curves of wearable sensor on the knee under motions of flexing/extending, walking, jogging, jumping, and squatting-jumping. “Adapted with permission from reference [65]. Copyright [2015], Advanced materials”. (**d**) Responsive curves of the graphene-silk fabric strain sensor to eyes blinking, mouth opening and closing, laughing and crying. “Adapted with permission from reference [79]. Copyright [2019], Advanced Materials Interfaces”. (**e**) Responsive curves of wearable sensors during the robot’s dance “Gangnam Style”: Elbow (black line), waist (redline), and knee (blue line). “Adapted with permission from reference [65]. Copyright [2015], Advanced materials”. (**f**) Relative resistance changes with various movements of the motion glove containing the fabric-based sensors, such as pressing, bending, grabbing, and up, down, and rotation of the wrist. “Adapted with permission from reference [83]. Copyright [2018], ACS Applied Materials & Interfaces”.

Based on the excellent performance of graphene-based textile strain sensors, they can be applied in various fields. Strain sensors are mainly used for detecting human motion. Human motions monitoring can be categorized as large human motions (walking, joint motion, finger bending, etc.) and subtle physiological motions (respiration, phonation, pulse wave, etc.) [45,46,50,51,52,57,58,59,61,62,64,65,66,68,70,71,72,76,78,79,80,83,84,85,86,87,88,91,94,95,96,97], as illustrated in Figure 4a–d. Graphene-based textile strain sensors are usually attached to different body parts, such as knees, fingers, elbows, eyes, mouth, etc., for sensing body movements. Cai et al. [97] attached a knitted fabric strain sensor to a body for monitoring human motions, including the flexion and rotation of the wrist and bending of fingers. Huang et al. [57] prepared a strain sensor, which can monitor a pulse wave, and eyeball movement, while keeping comfortable wearing sense. At present, most graphene-based textile strain sensors can be successfully applied to real-time monitoring of human motions. However, strain sensors are usually attached to body parts to sense movements of the human body, which are called “wearable”. It is still a challenge to develop stable and truly wearable graphene-based textile strain sensors.

Strain sensors can also be applied to complex robot detection and entertainment [65,77], as shown in Figure 4e. Cheng et al. [65] fabricated a yarn strain sensor, which not only can monitor a wide variety of human activities, including sleep, talk, pulse beat, walk, jog, jump, but also can detect and record of complex robot movements. Liu et al. [77] synthesized graphene woven fabric strain sensor, which can be used to create a wearable musical instrument. It allows the users to manipulate music through small body movements.

In addition, multiple strain sensors can be glued or woven into gloves to sense hand movements (Figure 4f) [83]. When the measured object moves, strain sensors deform. The electrical signal of sensors changes, resulting in sensing phenomenon.

## 5. Conclusions and Outlook

In this review, a variety of graphene-based textile strain sensors are evaluated comprehensively. The preparation methods, performance, and applications of textile strain sensors, including staple fiber, staple and filament yarn, nonwoven fabric, woven fabric, and knitted fabric strain sensors, are analyzed and discussed.

### 5.1. Challenges and Pitfalls

In spite of the graphene-based textile strain sensors having good performance, they face many challenges and pitfalls. First of all, the application capacity of graphene-based textile strain sensors is limited. Most researches only consider sensors water resistance, but do not consider the impact of other environmental factors, such as friction, PH, etc. Secondly, fiber/yarn strain sensors need flexible substrates such as PDMS [49,54,55,57,62,64,65], Ecoflex [58,59], or TPU [51] to protect their structure because sensing fibers and yarns are easily damaged, which may not achieve comfort in practical application. In addition, most sensors are attached to human skin to pick up signals. Although the sensitivity is high, the comfort is poor. Designing graphene-based fibers and yarns strain sensors with satisfactory comfort remains a big challenge. Thirdly, graphene-based textile strain sensors have good sensitivity and tensile properties, but it is difficult to achieve both high sensitivity and high tensile properties. Fourth, most of the resistance measurements are made using the two-probe method rather than the four-probe method, so that the contact resistance is included in the measurement resistance. During the strain process, both the contact resistance and sample resistance will change. The resistance obtained by using the four-probe method is more sensitive and more precise than that obtained by using the two-probe method, which can accurately reflect the damage of the sensing material caused by strain [117]. What is more, the nonlinear relationship prevails in textile strain sensors, but some articles do not properly deal with the nonlinear relationship between GF and strain [60,62].

### 5.2. Outlook

In general, graphene-based textile strain sensors can be obtained in two ways. One is to prepare conductive textiles through spinning and weaving techniques. The other is to deposit graphene-based materials on the surface of textiles. Due to the solubility and applicability of GO, most strain sensors are prepared by dip-coating method [64,65,66,67,68,69,70,87,88,89,90,91,92,93,94,95,96,97], which is simple and easy to operate regardless of the complexity of the preparation process, but the resulting pollution of GO manufacturing is of concern (That is, to prepare one gram of GO will consume at least 1000 g of water.).

Most of graphene-based textile strain sensors possess good sensitivity, stability, and sensing range. However, compared to staple fiber and filament strain sensor, fabric sensors attracted more attention in functionality and practical application, such as wear comfort [94], good wash fastness [72,80,85,97], easily integrating into clothes [71,79,84], self-cleaning, anti-corrosion ability [87], and waterproofness [83,87,91].

With the increase of strain range, the GF absolute value of graphene-based textile strain sensors usually presents two different variation trends. One is that GF decreases with the increase of strain [44,45,57,65,66,70]; the other is that GF increases with the increase of strain [59,68]. According to the structure of graphene-based textile strain sensors, when a small strain is applied, its GF value decreases with the increase of strain, which may be due to the decrease of contact points between fibers. When the strain exceeds the allowable range of fibers, the conductive networks of the textile surface break and irreversible damage occurs, leading to a sharp change in its resistance and a rapid rise in GF. Under a huge strain, the sensing material will crack or even fracture, leading to the infinite increase of GF. Therefore, the stretching stage of graphene-based textile strain sensors, which correspond to the structural change of textiles, can be judged according to the variation trend of GF value.

In addition, the nonlinear relationship prevails in textile strain sensors, although all the sensing linear relationship is within a certain range. It can be found that due to the slip inside the fiber or fabric and the change of textile strain sensor structure, the GF value of most textile strain sensors presents a nonlinear relationship with the strain. GF is the relative resistance that changes with strain. Therefore, the strain range applicable to GF values should be indicated.

In conclusion, in future studies, the effects of environmental factors such as friction and pH on the performance of graphene-based textile strain sensors are of concern. The nonlinear relationship of graphene-based textile strain sensors should be properly handled. Meanwhile, the GF value of strain sensors should be clearly analyzed to avoid misunderstanding. Moreover, in the test method, the four-probe method can be used more to measure resistance in order to avoid the impact of contact resistance on the experimental results.

## Figures and Tables

**Table 1:** Properties of various graphene-based staple fiber strain sensors.

Main Materials	Substrate	Fabrication Method	GF	Sensing Range (%)	Stability (Cycle)	Response Time (ms)	Application	References
Graphene	Graphene fiber	CVD	34.3–48.9 (8% strain)	-	-	-	-	[55]
RGO	Cellulose acetate fiber bundles	Dipping	−8.9 (<5% strain)	0.05–75	580 (0.3% strain)	-	Monitor full-range human motions	[64]
Graphene/ polyvinylidene fluoride/polyurethane	graphene fiber	Phase-separation	51 (0–5% strain) 87 (5–8% strain)	-	6000 (1% strain)	<100	Monitor a pulse wave, eyeball movement and joint motion	[57]
SBS/FLG	SBS/FLG composite fibers	Wet-spinning	160 (50% strain) 2546 (100% strain)	>110	-	-	-	[44]
SBS/graphene	SBS/graphene composite fibers	Wet-spinning	56 (0–40% strain) 1592 (40–73% strain) 10,084 (73–100% strain)	Up to 100	2100 (20% strain)	-	Detect human upper limb movements in different joints	[45]
RGO	RGO fibers	Wet-spinning/ vacuum filtration	1668 (~66% strain)	0.24–70	1200 (20% strain)	~30	Monitor fingers and arm movements, breathing during exercise and pulse be	[46]
RGO/TPU	RGO/TPU fibers	Electrospinning	~50 (50% strain)	0–150	500 (150% strain)	<160	Monitor full-range human motions	[50]

**Table 2:** Properties of various graphene-based staple and filament yarn strain sensors.

Main Materials	Substrate	Fabrication Method	GF	Sensing Range (%)	Stability (Cycle)	Response Time (ms)	Application	Other Advantages	References
Graphene/PVA	Graphene fiber	CVD/coating	~5.02 (1–6.3% strain)	Up to 16	200 (6% strain)	-	-	-	[54]
Carbon/graphene	Carbon/graphene composites nanofiber yarn	Electrospinning	>1700 (2% strain)	-	300 (2% strain)	-	Detect sound waves and tiny muscle movements	-	[51]
GO/ polyacrylonitrile	GO-doped polyacrylonitrile nanofiber yarn	Electrospinning	68 (0–20% strain)	0–100	>2000 (40% strain)	-	Monitor finger and neck movement	Can be woven into electronic fabric	[52]
GNPs/CB	Natural fiber yarn including flax and flax-bleached	Dip-coating	5.62 (4% strain)	Up to 60	>1000 (8% strain)	<209	Monitor various human movements	-	[58]
GNPs/CB/ conductive ink	Wool yarn	Dip-coating	5 (0–127% strain) 7.75 (127–200% strain)	Up to 200	1100 (75% strain)	~172	Detect various human movements	**-**	[59]
RGO	A double-covered yarn (PU core fiber and polyester fibers)	Dip-coating	10 (<1% strain) 3.7 (<50% strain)	Up to 100	10,000 (30% and 50% strain)	<100	Monitor a wide variety of human activities and complex robot movements	**-**	[65]
RGO	A double-covered yarn(~650 μm in diameter)	Dip-coating	8.8 (5% strain) 5.4 (10% strain) 1.6 (100% strain)	0–105	2000 (50% strain)	-	Monitor full-scale human motions	**-**	[66]
RGO	PU yarn	Dip-coating	50 (<50% strain) ~132 (90% strain)	0–90	30,000 (50% strain)	-	Monitor various human motions	Can be integrated into fabric structure	[68]
Graphene	Nylon filament	Dip-coating	0.08 (<12% strain) 0.07 (12–33% strain) 0.22 (33–45.69% strain)	Up to 45.69	2800 (40% strain)	-	Monitor human breathing and joint movement	-	[70]
Graphene/PVA	PU yarn	LbL	~87	-	100 (50% strain)	-	-	Thermal stability (within the range of 25–310 °C)	[60]
GMs/ Ag-nanoparticles	PU yarn	LbL	~500 (50% strain)	0–50	2000 (50% strain)	-	Monitor of human motions	Can be directly woven into textiles	[61]
GNSs/Au/ GNSs/PU	PU yarn	LbL	~662 (0–50% strain)	0–75	10,000 (50% strain)	-	Monitoring various human motions and control a hand robot	Waterproof property	[63]
GNP	RY	LbL	~1800	Up to 100	10 (80% strain)	-	Detect small-scale motions	-	[62]
GNP	NCRY	LbL	1.4	Up to 150	10 (100% strain)	-	Detect large-scale motion	-	[62]
GNP	WY	LbL	−0.1	Up to 50	10 (40% strain)	-	-	-	[62]

**Table 3:** Performance of various graphene-based nonwoven strain sensors.

Main Materials	Substrate	Fabrication Method	GF	Sensing Range (%)	Stability (Cycle)	Response Time (ms)	Application	Other Advantages	References
GO	Viscose nonwoven fabrics	Screen-printing	-	-	-	-	Detect the bending movements of wrist joint	Washing fastness (sheet resistance changes from ~1.6 to ~7.1 kΩ/sq after 5 washing cycles)	[85]
RGO	PET nonwoven fabric	Suction filtration	-	-	150 (10% strain)	-	Monitor human wrist movements	Electrothermal property (about 50 °C under a voltage of 6 V)	[86]
Graphene	Nonwoven fabric	Spray coating	9 (1% strain) 9.43 (20% strain)	-	20 (1–3% strain)	-	-	-	[73]
RGO	Nonwoven fabric	Dip-and-reduce	−7.1 (1% strain)	-	10,000 (1% strain)	-	Monitor human motions	-	[71]
RGO/carbon nanotubes	Nonwoven fabric	Dip-and-reduce	~33 (1% strain)	-	-	-	Monitor body motion and blood pulse	Machine washable (the resistance of RGO/CNTs/NWF textile changes from ~31 to ~36 kΩ after 6 washing cycles)	[72]
Graphene/ cellulose nanocrystal	TPU non-woven fabrics	Dip-coating	180 (15% strain)	Up to 98	>1000 (10% strain)	33	Detect full-range human activities	Self-cleaning, anti-corrosion ability and waterproofness (WCA = 154°)	[87]

**Table 4:** Performance of various graphene-based woven strain sensors reported in the literature.

Main Materials	Substrate	Fabrication Method	GF	Sensing Range (%)	Stability (Cycle)	Response Time (ms)	Application	Other Advantages	References
Graphene	Woven fabrics	CVD	~223 (3% strain)	-	1000	~72	A wearable musical instrument	-	[77]
Graphene	Woven fabrics	CVD	70 (10% strain) 282 (20% strain)	Up to 30	>4000 (5% and 50% strain)	~70	Detect physiological motions	-	[76]
GO	Polyimide fabric	Direct laser writing	27 (0–4% strain)	-	1000 (4% strain)	-	Detect human motion	-	[78]
RGO	Cotton bandage	Dip-coating	416 (0–40% strain) 3667 (48–57% strain)	Up to 57	>1000(0−7.5%, 0−15%, 0−30%, 0−45% strain)	<20	Monitor a wide range of human activities	-	[88]
Graphene	Cotton fabric	Dipping	2.49 (30% strain)	0–75	10,000 (30% strain)	~90	Detect the finger motion, book folding, ruler oscillating vibration, and phone speaker vibration	-	[82]
Graphene nanoplatelets/polyaniline	Lycra fabric	Spin-coating	~67 (0–40% strain)	Up to 40	1500 (5% and 9% strain)	-	Monitor the movement of five fingers	-	[17]
RGO	Cotton fabric	Dip and reduce	4 (3.3–5.5% strain)	-	100,000 (11.6% strain)	-	Monitor human movements	Waterproof properties	[83]
RGO	An elastic fabric composed of polyurethane and polyester	Dip-coating	4.13 (0–30% strain)	-	1000 (20% strain)	-	Monitor a full range of human activities	Water-resistant and skin-adhesive	[91]
RGO	Cotton fabric	Vacuum filtration	-	-	400	-	Monitor the wrist bending	Washability (sheet resistance changes from ~0.9 to ~1.2 kΩ/sq after 10 washing cycles)	[80]
RGO	Silk fabric	Vacuum filtration	124 (10% strain)	~10	1000 (2% strain)	-	Detect human motions	UV-blocking and hydrophobicity properties	[79]

**Table 5:** Properties of various graphene-based knitted strain sensors.

Main Materials	Substrate	Fabrication Method	GF	Sensing Range (%)	Stability (Cycle)	Response Time (ms)	Application	Other Advantages	References
RGO	Spandex/nylon knitted fabric	Dip-coating	~18.5 (<40.6% strain)	-	-	-	Monitor body movements	-	[94]
RGO	Weft-knit polyester fabric	Dip-coating	−1.7 (<15% strain, x-direction) −26 (<8% strain, y-direction)	Up to 50	500 (7.5% strain, x-direction) 500 (5% strain, y-direction)	-	Detect the physiological activity of human body	-	[95]
RGO	Polyester knitted elastic band	Dip-coating	34 (0–20% strain) 5 (20–50% strain)	0.2–50	>6000 (30% strain)	-	Monitoring of large scale as well as small-scale human body motions	-	[96]
RGO	Nylon/ polyurethane fabric	Dip-coating	18.5 (0–10% strain) 12.1 (10–18% strain)	0–33	120 (3% strain)	-	Monitor human motions	Washability (resistance increased from ~112 kΩ/m^2^ to ~154 kΩ/m^2^ after 8 washing cycles)	[97]
RGO	Wool-knitted fabric	Pad-dyeing	0.9 (20% strain, x-direction) 3 (20% strain, y-direction)	Up to 40	500 (20% strain, y-direction)	-	Detect human respiration movements and large motions	Seamlessly integrate with other fabric materials	[84]
